# Development and Implementation of Informational Toolkits to Address Inequities in COVID-19 Testing

**DOI:** 10.1089/heq.2025.0043

**Published:** 2025-06-06

**Authors:** Sunasia Mims, Jessica Rhinehart, Melissa Ryan, Susan Driggers, Travaé Hardaway Griffith, Grace Okoro, Tiffany Osborne, Lori Brand Bateman, Janet M. Turan, Raegan H. Durant, Barbara Hansen, Gabriela R. Oates

**Affiliations:** ^1^ Heersink School of Medicine, University of Alabama at Birmingham, Birmingham, Alabama, USA.; ^2^ School of Public Health, University of Alabama at Birmingham, Birmingham, Alabama, USA.

**Keywords:** COVID-19, testing, community engagement, informational toolkits

## Abstract

**Background:**

The COVID-19 pandemic disproportionately affected African American communities. Informational toolkits have emerged as a strategy to address such inequities. Community-driven approaches to toolkit design may enhance their relevance and impact in underserved communities.

**Methods:**

Using a human-centered design approach, we developed COVID-19 testing toolkits tailored to health care, faith-based, and public housing settings in rural and urban communities. A group of community stakeholders representing each setting was recruited to co-create the toolkits. The design process began with an intensive two-day workshop to deliberate on content, format, and dissemination channels, followed by virtual meetings and iterative prototyping cycles that incorporated stakeholder feedback. Given the complexity of implementing such toolkits in health care settings, additional measures were taken to support and assess implementation at the participating health facility sites.

**Results:**

The toolkits included core resources, such as training modules, testing guidelines, and maps, and setting-specific content, such as appointment reminders, pulpit announcements, and emergency contact sheets. Materials were provided in both digital and print formats. Onboarding and technical training facilitated implementation in health care settings. Pre/post implementation surveys showed high perceived usefulness and feasibility of the health care toolkits. Implementation patterns favored print resources, with appointment reminders being most utilized. Leadership support enhanced toolkit credibility and adoption. Implementation challenges included COVID-19 fatigue, technology limitations, and leadership transitions.

**Conclusion:**

Informational toolkits co-developed with community stakeholders provide a model for translating research into solutions that enhance health equity. Prioritizing community perspectives can improve preparedness for future crises. Successful implementation requires adaptability, multimodal delivery, and sustained leadership buy-in.

## Introduction 

The COVID-19 pandemic disproportionately affected low-resource communities and marginalized racial groups in the United States,^1,2^ with African Americans shouldering a higher burden of the morbidity and mortality from the virus.^3–5^ The spread of the aerosolized virus rapidly increased the demand for testing services,^6^ making accurate testing information and access to testing critical. Access to COVID-19 testing was particularly important among vulnerable populations due to their disproportionate representation in service sector jobs^7^ deemed as essential to the country.^8^ However, multiple barriers, such as a lack of convenient testing sites, limited transportation, confusion about testing eligibility, and misperceptions of cost, made testing difficult.^9,10^ These barriers were compounded by fears of lost wages or employment due to a positive test, as well as by experiences of COVID-19 stigma.^11,12^

Informational toolkits have emerged as an important mechanism for translating research into practice in a variety of settings, including clinical care,^13–15^ nursing homes and long-term care facilities,^16,17^ homeless shelters,^18^ and schools.^19^ Such toolkits typically consist of action-oriented guidelines and resources that help bridge the gap between evidence-based knowledge and real-world implementation in health care, education, and social services fields.^15^

Toolkit designers must navigate several challenges, including balancing generalizable resources with user needs, honoring community expertise while communicating evidence-based practices, and creating materials that are both comprehensive and user-friendly. The development process often involves participatory approaches, engaging stakeholders through focus groups, workshops, and co-design sessions to ensure relevance, usability, and adaptation. Community-driven informational toolkits that directly involve the affected communities in the co-creation and implementation of the final product are particularly impactful.^20,21^ Grounding the toolkit development process in community and stakeholder insights results in an informative, culturally relevant, and useful product^22,23^ with a high potential for reducing health disparities.^22^

To that end, the Reducing Ethical and Social Prejudicial Effects of COVID-19 Testing in Underserved Populations (RESPECT-UP) study used a Human Centered Design (HCD) approach and engaged community stakeholders throughout the process to develop a compendium of COVID-19 testing resources that were organized into informational toolkits tailored to various community settings. The toolkits aimed to facilitate equitable, non-stigmatizing, and non-discriminatory COVID-19 testing in vulnerable urban and rural African American communities in Alabama and similar settings in the U.S. South. They also served as a model for ensuring equitable testing in the event of future viral outbreaks or pandemics.

## Methods

The RESPECT-UP study is described in detail elsewhere.^11,12^ Briefly, we recruited African American residents of Census tracts in the top 10th percentile of the Social Vulnerability Index (SVI)^24^ in two Alabama counties—Jefferson (urban) and Dallas (rural). Using an explanatory sequential mixed methods study design, we collected quantitative (surveys) and qualitative data (focus groups, key informant interviews) to assess social, ethical, and behavioral determinants of COVID-19 testing in the identified communities. Findings from this mixed-methods study informed the development of the RESPECT-UP toolkits. The study was approved by the Institutional Review Board at UAB (protocol number IRB-300008595).

The toolkit development was grounded in a human-centered design (HCD) approach,^25^ which emphasizes participatory and iterative design process and understanding and addressing the specific needs of users.^25^ It also integrates what is desirable from a human point of view with what is economically viable and technologically feasible.^26,27^ The HCD process often lasts between 2 and 3 months and consists of three phases, including inspiration, ideation, and implementation. We used rapid prototyping, which tests multiple toolkit solutions with real users in short iteration cycles before committing to a single solution. The specific HCD strategies used for developing the RESPECT-UP toolkits were unique to the targeted communities and settings.

As suggested by Kia-Keating et al.,^28^ the toolkit development team was recruited to represent varieties of backgrounds and experiences and include potentially opposing perspectives. We focused on three community settings: health care, faith-based organizations, and public housing. Participants were community stakeholders identified by the RESPECT-UP Community Advisory Board and through existing community partnerships. They had to be adults residing in Jefferson or Dallas County and representing one of the three community settings above, agree to participate in at least two in-person toolkit development workshops in addition to virtual sessions, and be available to facilitate implementation of the toolkits in their respective settings.

Toolkit development began with an intensive two-day in-person workshop facilitated by the RESPECT-UP communication and community engagement teams. The workshop included an introduction to the fundamentals of toolkit development, followed by a presentation of the RESPECT-UP integrated quantitative and qualitative findings in urban and rural settings. Workshop facilitators used a topic guide and conversation starters to generate discussions about COVID-19 testing. Participants then separated into setting-specific breakout sessions to deliberate on toolkit content, format, and dissemination channels and made setting-specific recommendations. Each session featured facilitators and note takers and was audio-recorded to ensure accurate documentation of the conversations. After the workshop, all session notes and recordings were transcribed, and common themes were identified to establish foundational components for toolkit development.

Four weeks after the intensive in-person workshop, a virtual meeting with all participants was convened to communicate workshop findings and verify the accuracy of information. This meeting also provided stakeholders with an opportunity to elaborate on their suggestions and introduce new ideas. Using virtual breakout rooms and on-screen polls, participants ranked the types of proposed resources on their perceived effectiveness for the respective settings. Afterwards, multiple toolkit versions were prototyped in short iterative cycles based on feedback received through email correspondence, one more in-person session, and two virtual sessions, before the final versions of RESPECT-UP toolkits were developed.

To inform implementation, the health care toolkit was deployed and evaluated in one urban and one rural primary care clinic. Given the complexity of implementing such toolkits in health care settings, additional measures were taken to support implementation at the participating health facility sites. These included orientation sessions for staff on toolkit content and training on dissemination protocols to ensure standardized implementation. Fidelity, or the degree to which intervention is implemented as intended, was evaluated with a checklist completed by implementers (health care providers and administrators).

Using surveys with implementers before and after the toolkit deployment, we assessed implementation outcomes suggested by Proctor’s Conceptual Model of Implementation Research.^29^ Feasibility, or the extent to which intervention can be implemented in the setting, was evaluated with the Feasibility Measure (FIM);^30^ acceptability, or the perception among implementers that intervention strategies are satisfactory, was evaluated with the Acceptably Measure (AIM);^30^ and appropriateness, or the perceived fit of intervention to the setting, was evaluated with the Appropriateness Measure (IAM).^30^ Each instrument included four questions, with response options on a 1–5 Likert scale (1 = Completely disagree, 2 = Disagree, 3 = Neither agree or disagree, 4 = Agree, 5 = Completely agree). Staff ranked seven toolkit elements (training materials, community resources, symptom quiz, educational videos, resource business card, schedule reminder template, and door flyer) based on usefulness (1–7 scale, from most to least useful). The post-implementation survey included additional items asking about website access frequency and open-ended questions about implementation experience. Finally, a post-implementation virtual focus group with implementers at the urban site was conducted via Zoom. Prompts assessed six implementation domains: acceptability, adoption, appropriateness, utilization, penetration, and sustainability. Sessions were recorded, transcribed, and analyzed using thematic approach.

## Results

The toolkit development team consisted of 15 community stakeholders, with 5 representatives from each of the three community settings: health care, faith-based organizations, and public housing, spanning both rural and urban areas. The stakeholders included individuals with experience as health care providers, pastors, and housing managers. The toolkit development process lasted three months, and the toolkits were housed on the RESPECT-UP website.^31^

### Common and Tailored Content

The toolkits included core (common) content and tailored content customized to the unique needs of the three types of settings. Common elements included training modules, testing guidelines and best practices, informational resources, a guidebook with context-specific recommendations, maps with recommended locations of community testing sites, and up-to-date local resources for post-testing support. Each setting requested specific content tailored to their respective communities, such as appointment reminders for health care, pulpit announcements for faith-based organizations, and informational magnets for public housing.

### Digital and Printed Resources

The toolkits included both digital and printed resources designed for diverse age groups and varying levels of technological proficiency. Hosted on a website hub,^31^ the digital content included a COVID-19 symptom calculator; the latest CDC guidelines for COVID-19 testing, quarantine, and vaccination; local information on meeting basic needs, such as food, housing, and transportation assistance; training modules on bias, belonging, empathy, mindfulness, burnout, stress, cultural competency, and conflict resolution; and a COVID-19 “Jeopardy” game. The digital resources complemented the printed resources by providing in-depth information and interactive elements that can be easily updated as guidelines and recommendations change.

Printed resources included setting-specific flyers, posters, magnets, cards, pulpit announcements, and emergency contact sheets as described below.

#### Health care

**FIG. 1.**
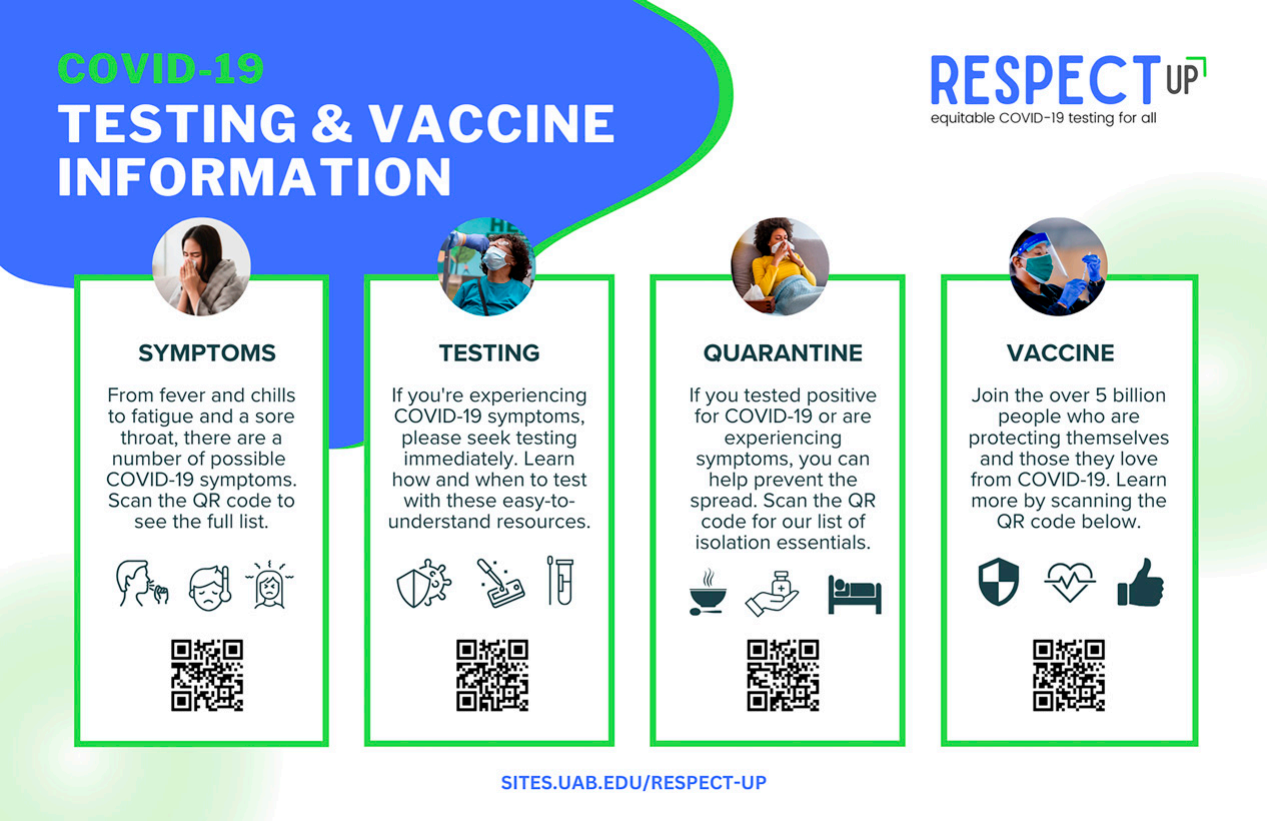
RESPECT-UP COVID-19 testing informational poster. RESPECT-UP, Reducing Ethical and Social Prejudicial Effects of COVID-19 Testing in Underserved Populations.

The health care toolkit included a multipage website, 8.5″ × 11″ and 11″ × 17″ COVID-19 resource posters (Fig. 1), 4″ × 6″ COVID-19 resource cards, refrigerator magnets, and appointment reminder sheets. The digital and print products included QR codes for easy access to the RESPECT-UP website. With approval from stakeholders, the RESPECT-UP team conducted site visits to implementation locations (local hospitals) to determine the optimal placement for toolkit components, enhancing the overall dissemination strategy.

#### Faith-based

**FIG. 2.**
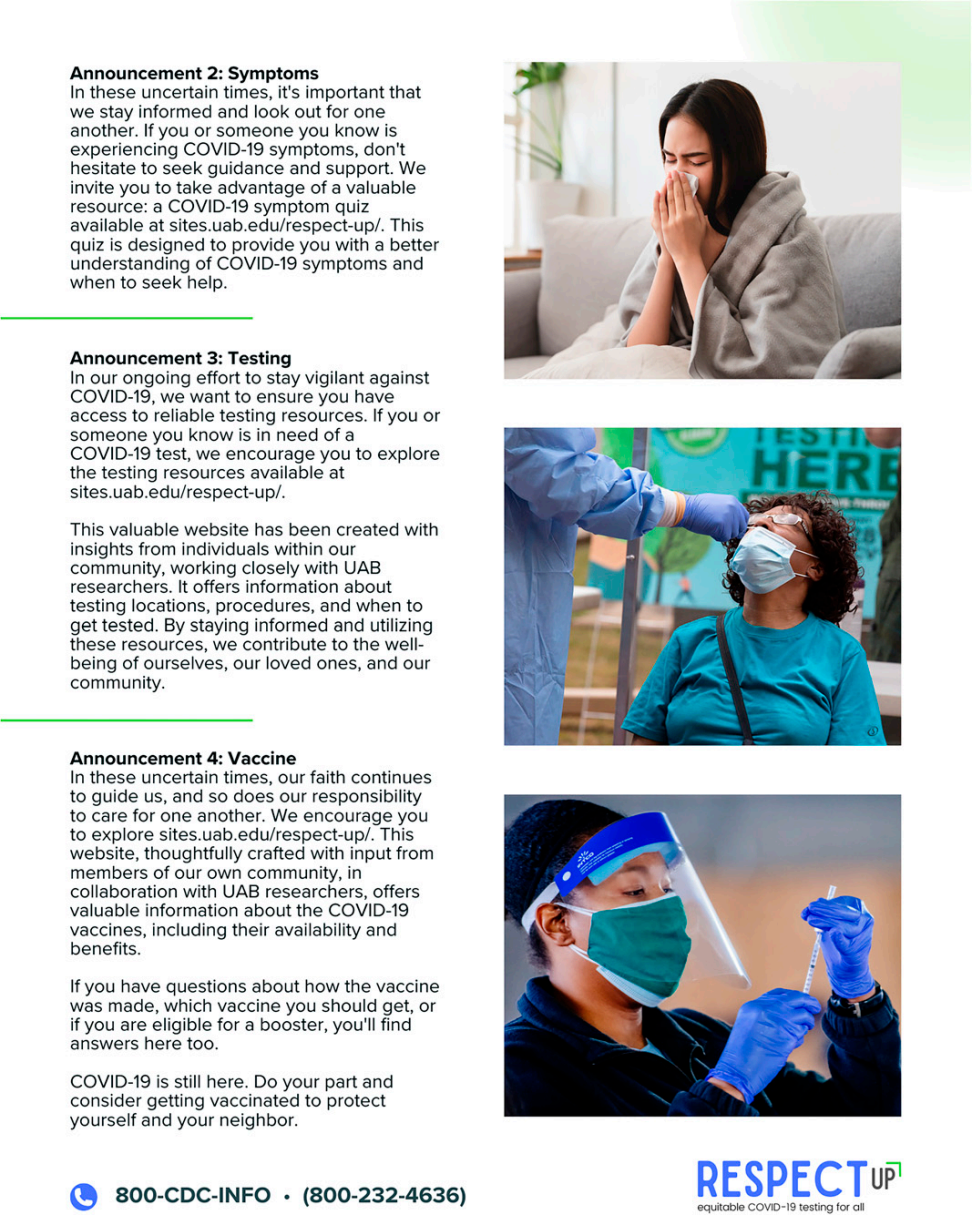
RESPECT-UP COVID-19 testing pulpit announcements. RESPECT-UP, Reducing Ethical and Social Prejudicial Effects of COVID-19 Testing in Underserved Populations.

Stakeholders from faith-based settings emphasized the role of faith leaders in guiding their communities and the need for resources to help explain the importance of COVID-19 testing. The faith-based toolkit, therefore, included sample COVID-19 pulpit announcements (Fig. 2), 8.5″ × 11″ and 11″ × 17″ COVID-19 resource posters, and a business card-sized COVID-19 resource. All materials feature QR codes to the RESPECT-UP website and a phone number to the Centers for Disease Control and Prevention (CDC) for individuals preferring direct contact.

#### Public housing

**FIG. 3.**
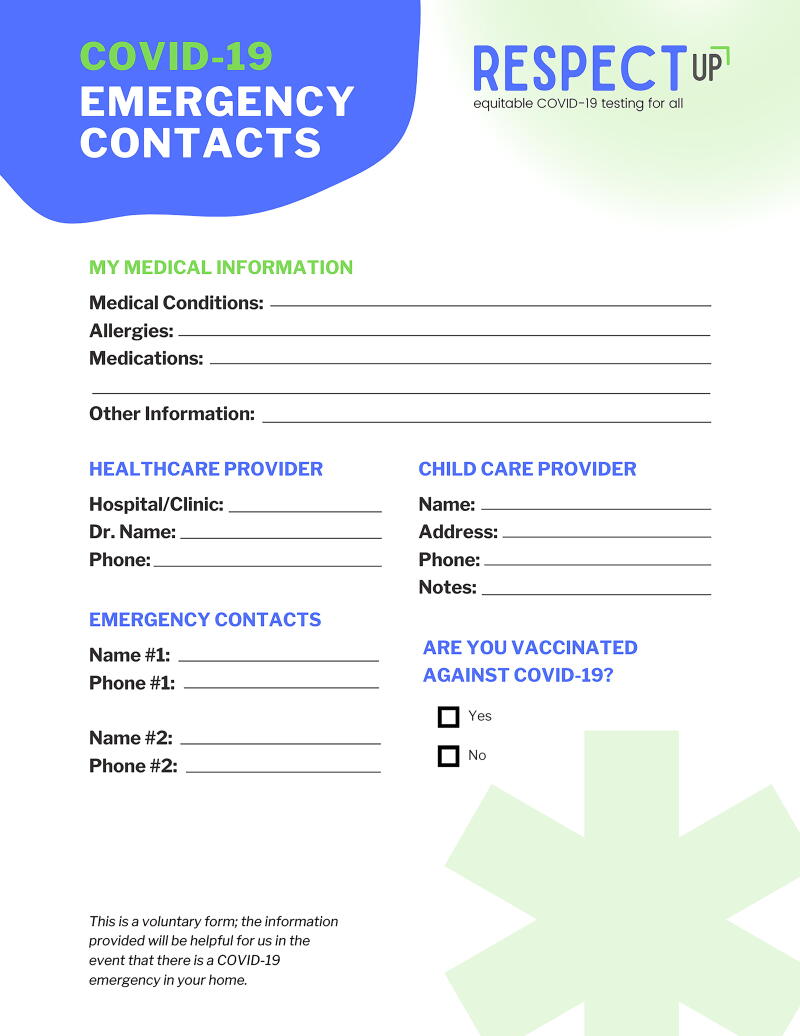
RESPECT-UP emergency contact information sheet for public housing communities. RESPECT-UP, Reducing Ethical and Social Prejudicial Effects of COVID-19 Testing in Underserved Populations.

Stakeholders from public housing settings highlighted the need for efficient identification emergency and next of kin contacts. Thus, the public housing toolkit included a COVID-19 emergency contact sheet, refrigerator magnet with resource information, and 8.5″ × 11″ and 11″ × 17″ COVID-19 resource posters (Fig. 3). As housing units are equipped with refrigerators, the magnets were the preferred medium for information about local COVID-19 resources.

### Implementation in Health Care Settings

Implementation began with a structured orientation session at each health care site. In these sessions, we briefed health care staff on the study aims, study findings, and the toolkit development process. We then conducted technical training on toolkit content and dissemination protocols. An environmental assessment by study staff was conducted to optimize material placement and establish dissemination workflows.

Pre-implementation surveys with 19 health care providers and staff (*n* = 10 at the urban site, *n* = 9 at the rural site) showed positive responses to the toolkit (68% “Completely agree” at the urban site, 74% “Completely agree” at the rural site); no respondents selected “Disagree” or “Completely disagree.” Staff perception of implementation feasibility was high, with 70% of urban site respondents and 56% of rural site respondents indicating they “Completely agree” that the toolkit was implementable, possible, and doable. The evaluation of toolkit components showed different patterns of perceived usefulness between sites. At the rural site, most components were consistently rated as “Most Useful” by the majority of respondents (5 out of 9), with training materials, symptom quiz, and educational videos receiving the highest ratings. At the urban site, ratings were more varied. Training materials were rated as “Most Useful” by 6 out of 10 respondents, while other components received mixed ratings. The appointment reminder cards and website resources were selected as most promising for patient engagement at the rural site, while the urban site showed more varied preferences for engagement tools.

Post-implementation surveys (*n* = 8) reported toolkit utilization patterns that differed from the pre-implementation assessments. The survey results were confirmed by focus group participants (*n* = 4). Both survey and focus group data indicated that while staff successfully implemented the toolkit, actual usage varied by the site’s organizational and technological capabilities. Appointment reminders were the most used tool, and focus group participants specifically noted the effectiveness of these materials for patient engagement. Although the urban site was able to integrate toolkit materials into their electronic system for text messages and emails, technological limitations at both clinics restricted the use of digital resources. For example, the absence of digital signage in waiting rooms and common areas led to greater reliance on printed materials. Staff reported using a combination of printed materials, including appointment reminders, posters, and handouts. Website and other electronic resources were used less frequently than anticipated in pre-implementation assessments.

Thematic analysis of focus group discussions revealed six themes: toolkit utilization, technology limitations, COVID-19 fatigue, effectiveness of printable materials, importance of leadership support, and perceived risk and motivation for testing. Several implementation challenges and contextual factors affecting the toolkit’s impact were identified. Notably, participants observed increasing COVID-19 fatigue as the pandemic progressed, with patients becoming more complacent about testing and vaccination, which reduced the perceived urgency of the intervention. Patient motivation for testing was often linked to perceived risk, with individuals more likely to seek testing when planning to be around vulnerable individuals. Leadership support emerged as a critical factor in successful toolkit implementation: for example, the endorsement of the toolkit by the medical director significantly enhanced its credibility and integration into clinical practice. Implementation challenges were particularly notable at the rural site, where a change in clinical leadership during the study period negatively impacted the timeline and reach of toolkit adoption. This leadership transition affected staff availability for training and reduced institutional bandwidth for incorporating new protocols, thereby highlighting the critical role of leadership buy-in for the successful implementation of interventions in clinical settings.

## Discussion

To facilitate equitable, non-stigmatizing, and non-discriminatory COVID-19 testing in vulnerable urban and rural African American communities and provide a template for equitable testing in future viral outbreaks and pandemics, we used a community-engaged HCD approach to develop a compendium of COVID-19 testing resources organized into informational toolkits tailored to three types of community settings: public housing, faith-based, and health care.

The RESPECT-UP toolkits demonstrated the potential of community-driven approaches for addressing health disparities, particularly in the context of the COVID-19 pandemic. Engaging stakeholders from various settings in all stages of the design process, from ideation to implementation, produced toolkits that were informative, culturally relevant, and practical.

While toolkits facilitate knowledge transfer among researchers, practitioners, and community members, the community-driven process of developing them is impactful in its own right. Direct involvement of community stakeholders can empower communities to address issues like misinformation, digital divide, and health equity.

The approach used in development of the RESPECT-UP COVID-19 testing toolkits was similar to the one described by Wilner et al.^32^ regarding the development of toolkits for advocacy work. Connecting with the user on a human-centered level can help them connect with the message.^32^ The use of an HCD approach also allowed us to tailor the toolkits to the specific needs and preferences of each type of community setting. For example, the emphasis on pulpit announcements in the faith-based toolkit reflected the important role of religious leaders as trusted sources of information in their communities.^2,33^ The inclusion of emergency contact sheets in the public housing toolkit addressed a need identified by stakeholders in that setting, whereas the appointment reminder sheets met a stated need of stakeholders from health care settings.

One of the key strengths of the toolkits was their focus on multiple levels of influence, in a social-ecological framing.^34^ On an individual level, the toolkits addressed a person’s knowledge, attitudes, and behaviors; on an interpersonal level, they focused on the network of family, friends, and coworkers; on an organizational level, they considered the needs of institutions such as churches, health care facilities, and public housing; on the community level, they addressed cultural norms and responded to the physical environment, including services and resources; and on the policy level, they focused on local, state, and federal COVID-19 policies and guidelines. By providing resources that address both immediate informational needs (COVID-19 symptoms and testing guidelines) and broader unmet social needs (access to food and housing assistance), toolkits such as ours have the potential to make a meaningful impact on health equity.

It is important to acknowledge that toolkit implementation faces several challenges. Ensuring consistent use across various settings and maintaining the toolkits’ relevance in a rapidly changing pandemic context requires both ongoing engagement with stakeholders and regular updates to the resources. As Davis et al. noted, “unless the toolkit is used, it won’t help solve the problem.”^35^

Implementation success was heavily dependent on both technological infrastructure and sustained leadership support across settings. While the toolkit was designed to include both digital and print resources, technological limitations in some clinical settings restricted the full utilization of digital components, forcing greater reliance on print materials. This experience highlighted the importance of maintaining flexible, multimodal delivery options to accommodate varying levels of technological capacity across implementation sites. Leadership engagement emerged as another critical factor, as demonstrated by the contrasting experiences at the two sites. Strong leadership endorsement at the urban site enhanced toolkit credibility and facilitated integration into clinical workflows, while leadership transitions at the rural site disrupted implementation momentum and reduced institutional capacity for adoption.

The implementation process also revealed shifting contextual challenges that affected toolkit utilization. As the pandemic evolved, growing COVID-19 fatigue among both staff and patients decreased engagement with toolkit resources, particularly for testing promotion. This temporal dimension of implementation highlighted the need for toolkit content and messaging to remain adaptable to changing circumstances and public health priorities. Additionally, the notable preference for appointment reminders over other toolkit components suggested that successful implementation may depend less on comprehensive resource utilization and more on identifying and emphasizing the specific tools that resonate most strongly with each setting’s workflow and user needs.

### Health Equity Implications

The RESPECT-UP toolkits were designed to enhance access to and uptake of COVID-19 testing and help facilitate equitable, non-stigmatizing, and non-discriminatory testing in future viral outbreaks in vulnerable urban and rural communities. Prioritizing community perspectives in public health interventions can result in more engaged communities that are better prepared to face health crises. Informational toolkits co-developed with community stakeholders offer a template for translating research into community-driven and community-serving solutions that improve the health of all.

## Abbreviations Used

CDC: Centers for Disease Control and Prevention
HCD: Human-centered design
RESPECT-UP: Reducing Ethical and Social Prejudicial Effects of COVID-19 Testing in Underserved Populations
UAB: University of Alabama at Birmingham
